# *In Vitro* Design and Evaluation of Phage Cocktails Against *Aeromonas salmonicida*

**DOI:** 10.3389/fmicb.2018.01476

**Published:** 2018-07-06

**Authors:** Ling Chen, Shengjian Yuan, Quan Liu, Guoqin Mai, Jinfang Yang, Deng Deng, Bingzhao Zhang, Chenli Liu, Yingfei Ma

**Affiliations:** ^1^Institute of Synthetic Biology, Shenzhen Institutes of Advanced Technology, Chinese Academy of Sciences, Shenzhen, China; ^2^College of Life Sciences and Oceanography, Shenzhen University, Shenzhen, China; ^3^R&D Center, Shenzhen Alpha Feed Co., Ltd., Shenzhen, China

**Keywords:** *in vitro*, *Aeromonas salmonicida*, therapeutic agents, phage cocktails, *T4virus*

## Abstract

As an alternative approach against multidrug-resistant bacterial infections, phages are now being increasingly investigated as effective therapeutic agents. Here, aiming to design an efficient phage cocktail against *Aeromonas salmonicida* infections, we isolated and characterized five lytic *A. salmonicida* phages, AS-szw, AS-yj, AS-zj, AS-sw, and AS-gz. The results of morphological and genomic analysis suggested that all these phages are affiliated to the *T4virus* genus of the *Caudovirales* order. Their heterogeneous lytic capacities against *A. salmonicida* strains were demonstrated by experiments. A series of phage cocktails were prepared and investigated *in vitro*. We observed that the cocktail combining AS-gz and AS-yj showed significantly higher antimicrobial activity than other cocktails and individual phages. Given the divergent genomes between the phages AS-yj and AS-gz, our results highlight that the heterogeneous mechanisms that phages use to infect their hosts likely lead to phage synergy in killing the host. Conclusively, our study described a strategy to develop an effective and promising phage cocktail as a therapeutic agent to combat *A. salmonicida* infections, and thereby to control the outbreak of relevant fish diseases. Our study suggests that *in vitro* investigations into phages are prerequisite to obtain satisfying phage cocktails prior to application in practice.

## Introduction

*Aeromonas salmonicida* is a waterborne pathogenic bacterium considered to be one of the most relevant fish pathogens and is the causative agent of furunculosis in aquaculture systems worldwide (Janda and Abbott, 2010; Austin and Austin, 2016; Menanteau-Ledouble et al., 2016). Recently, efforts to control furunculosis rely heavily on antibiotics or vaccines (Bergh et al., 2013; Dallaire-Dufresne et al., 2014; Coscelli et al., 2015; Austin and Austin, 2016). However, the intensive use of antibiotics in treating furunculosis has driven the attendant development of multidrug-resistance in *A*. *salmonicida* (Cabello, 2006; Defoirdt et al., 2007; Reith et al., 2008; Kim et al., 2011; Austin and Austin, 2016) and has now become a global concern (Tanji et al., 2005; Barbu et al., 2016).

Bacteriophages (phages) are natural viruses that specifically infect bacteria (Chan et al., 2013). To date, several studies have demonstrated the potential of using phages to treat infections caused by multidrug-resistant bacteria (Hagens and Loessner, 2007; Labrie et al., 2010; Pereira et al., 2011; Kim et al., 2012b; Chan et al., 2013; Watkins et al., 2014; Pereira C. et al., 2016; Silva et al., 2016). Nonetheless, the host range of a single phage type tends to be relatively narrow, often consisting of only a subset of strains of a given bacterial species. This extreme specificity severely limits the use of only one phage type to control bacterial infections. Moreover, using only a single phage type to control a bacterial infection can also readily drive the emergence of phage-resistant bacterial mutants (Hyman and Abedon, 2010; Pirnay et al., 2011; Chan et al., 2013; Castillo et al., 2015; Melo et al., 2016; Yen et al., 2017).

The limitations of single phage application led to the idea of using phage cocktails. The development of phage cocktails comprised of multiple, different phage types can potentially circumvent the development of phage-resistant bacterial mutants and broaden the host range (Wright et al., 2009; Kelly et al., 2011; Jaiswal et al., 2013; Mapes et al., 2016) used six phages to treat *Pseudomonas aeruginosa* associated chronic otitis, which conferred significant improvement in clinic. Mateus et al. (2014) applied a cocktail containing two or three phages to control fish diseases, and the result confirmed that the use of phage cocktails increased the efficacy of phage therapy in inactivation of *Vibrio* spp. (Mateus et al., 2014). Nevertheless, including too many phages in a cocktail will drive up development and manufacturing costs (Hyman and Abedon, 2010; Pirnay et al., 2011; Stephen and Abedon, 2012; Barbu et al., 2016).

Here, we isolated five lytic bacteriophages infecting *A. salmonicida* from seawater in China and characterized their morphology, genomes, and infection cycles. We grouped the five phages into various combinations (cocktails) and assessed their ability in inhibiting *A. salmonicida* growth *in vitro*. The results suggest that the most efficient combination consists of two phages. Our optimized phage cocktail was capable of inactivating the fish pathogen over a period of 80 h and displayed higher efficacy, broader temperature, and pH adaptation ranges against the pathogen than other phage cocktails designed in this study. Taken together, our study shows that the two-phage cocktail is a promising agent for the management of *A. salmonicida* infections in aquaculture.

## Materials and Methods

### Bacterial Strains and Culture Conditions

All of the bacterial strains used in this study were verified by 16S rRNA gene sequencing or complete genome sequencing (**Table 1** and Supplementary Table S1). The *A. salmonicida* strains used in this study were isolated from the intestinal homogenates of various diseased fishes collected from different geographical locations in China. *A. salmonicida* (KC254648.1 and KC254649.1) were purchased from the State Key Laboratory of Freshwater Ecology and Biotechnology (Institute of Hydrobiology, Chinese Academy of Sciences, Wuhan, China) (Long et al., 2016). *A. salmonicida* (CP022175.1, CP022186.1, and CP022181.1) were provided by the State Key Laboratory of Microbiology Resources (Institute of Microbiology, Chinese Academy of Sciences, Beijing). *A. salmonicida* (MF632072) was provided by Sichuan Agricultural University (Chengdu, Sichuan, China). *A. hydrophila* strains (MF663676, MF663672) were isolated from the wastewater of an aquaculture farm in Zhanjiang City, Guangdong Province. The rest of the strains were isolated from various environmental samples. All of the strains were stored at -80°C and fresh plated bacterial cultures were maintained for less than 3 days in solid LB medium at 4°C prior to conducting experiments (Pereira S. et al., 2016). One isolated colony was aseptically transferred to LB medium and cultured overnight. All cells were collected at the exponential stage (OD_600_ = 0.2–0.6). We collected water samples that putatively contain *Aeromonas* phages from various locations in Guangdong Province, China. With the purpose of developing phage antimicrobial agents against the pathogen of *A. salmonicida* occurring in aquaculture in South of China, we selected 30°C as the operating temperature in this study.

**Table 1:** Phage lytic spectra on the bacterial strains used in this study.

Species	Infectivity						
	Accession	AS-szw	AS-yj	AS-zj	AS-sw	AS-gz	Cocktail
*Aeromonas salmonicida* strain^∗^	MF663675.1^e^	+	+	+	+	+	+
*A. salmonicida BG* strain^a^	KC254648.1^f^	+	+	+	+	+	+
*A. salmonicida YK* strain^a^	KC254649.1^f^	+	+	+	+	+	+
*A. salmonicida S121* strain^b^	CP022175.1^f^	+	+	+	+	+	+
*A. salmonicida S68* strain^b^	CP022186.1^f^	+	+	+	+	+	+
*A. salmonicida S44* strain^b^	CP022181.1^f^	+	+	+	+	+	+
*A. salmonicida* strain^c^	MF632072^e^	+	+	+	+	+	+
*A. hydrophila* strain^d^	MF663676^e^	-	-	+	-	-	+
*A. hydrophila* strain^d^	MF663672^e^	-	-	+	+	+	+

^∗^*Strain isolated in aquaculture in South of China and used on the enrichment procedure for phage isolation. ^*a*^Strains purchased from State Key Laboratory of Freshwater Ecology and Biotechnology, Institute of Hydrobiology, Chinese Academy of Sciences, Wuhan. ^*b*^Strains gained from State Key Laboratory of Microbiology Resources, Institute of Microbiology, Chinese Academy of Sciences, Beijing. ^*c*^Strain provided by Sichuan Agricultural University (Chengdu City, Sichuan Province). ^*d*^Strains isolated from wastewater of an aquaculture farm in Zhanjiang City, Guangdong Province. ^*e*^16S rRNA sequences number of the relative strains waiting to be released. ^*f*^Genome sequences number of the relative strains*.

We typed the seven *A. salmonicida* strains based on the methods reported by Szczuka and Kaznowski (2004). Briefly, random amplified polymorphic DNA PCR (RAPD-PCR) method with primers AP5 (5′-TCACGCTGCG-3′) (Talon et al., 1998), OPB-7 (5′-GGTGACGCAG-3′) (Oakey et al., 1996), and AP3 5′-TCACGATGCA-3′ (Talon et al., 1998) and enterobacterial repetitive intergenic consensus sequence PCR (ERIC-PCR) method with primers ERIC-R (5′-ATGTAA GCTCCTGGGGATTCAC-3′) and ERIC2 (5′-AAGTAAGTGACTGGGGTGAGCG-3′) (Versalovic et al., 1991) were employed to type the subspecies of the *A. salmonicida* strains used in study. RAPD-PCRs were carried out as previously described with primers AP5, OPB-7, and AP3, respectively (Szczuka and Kaznowski, 2004). The PCR program was: 1 min at 94°C, 25 cycles of 1 min at 94°C, 1 min at 36°C, and 2 min at 72°C, then 15 cycles of 1 min at 94°C, 1 min at 36°C, and 3 min at 72°C, and the last step for 2 min at 72°C for final extension. ERIC-PCR was performed using primers ERIC-1R and ERIC2 (Szczuka and Kaznowski, 2004). The PCR program was: 7 min at 95°C, then 30 cycles of 30 s at 90°C, 1 min at 52°C, and 8 min at 65°C, and the last step for 16 min at 65°C. The patterns of the PCR products were visualized by 1.5% agarose gel (wt/vol).

Susceptibility test of *A. salmonicida* YK and BG on 24 antibiotics was carried out according to the methods described by Vlková et al. (2006) and Liasi et al. (2009).

### Bacteriophage Isolation, Purification, and Host Range Determination

*Aeromonas salmonicida* (MF663675) was used as a host to screen the phages. Phage isolation was carried out according to the method reported by Pereira et al. (2011), with minor modifications. Briefly, the water samples from several locations in China were centrifuged at 8000 × *g* for 10 min to remove the solid impurities, and the supernatants were filtered through a membrane filter (0.22 μm) to remove bacterial debris. To enrich the phages, 100 ml aliquots of the filtrates were mixed with 50 ml of *A. salmonicida* (MF663675) cultures and grown at 30°C overnight. The resulting cultures were centrifuged at 10,000 × *g* for 10 min, and the supernatant was filtered using 0.22 μm to remove residual bacterial cells. Next, the filtrate containing enriched phages (0.1 ml) was mixed with 0.5 ml of the host cells (OD_600_ = 0.4–0.9) in LB medium and 5 ml of molten top soft nutrient agar (0.7%). The mixture was overlaid onto solidified base nutrient agar (1.5%). After incubation for 4–8 h at 30°C, clear phage plaques were picked from the plate. Phages were purified according to the standard methods (Gencay et al., 2017). Phage titers were determined with the double-layered method. The isolated bacteriophages were stored in 20% glycerol at -80°C.

The host range of the phages was determined by spot testing on a panel of 38 strains belonging to different genera (listed in Supplementary Table S1). Briefly, plates prepared as described above using the strains listed in Supplementary Table S1 were incubated at 30°C and plaques were examined after 8–12 h. Bacterial sensitivity to a given bacteriophage was evaluated on the basis of the lysis-cleared zone at a spot: bacteria were differentiated into two categories according to the clarity of plaques: lysis (+) and no lysis (-). Additionally, *A. salmonicida* strains YK and BG incubated at 18°C were also used for host range determination.

### Transmission Electron Microscopy of Phages

Purified phage particles from a highly concentrated suspension (10^9^ PFU/ml) in SM buffer [100 mM NaCl, 8 mM MgSO_4_, 5 mM Tris–HCl (pH 7.5), 0.01% gelatin] were adsorbed onto carbon-coated copper grids, and then were negatively stained with 2% phosphotungstic acid (pH 7.0). After drying at room temperature, the grids were examined using TECNAI G2 F20 S-Twin transmission electron microscope (FEI, United States).

### Genome Sequencing, Assembly, and Annotation

Bacteriophage particles were purified by discontinuous CsCl centrifugation, and genomic DNA was extracted using a lambda bacteriophage genomic DNA Rapid extraction kit (DN22; Aidlab, China) following the manufacturer’s protocol. The DNA samples were visualized on 0.7% agarose gels containing SYBR^TM^ safe DNA gel stain (Invitrogen, United States), and the extracted phage DNA was sequenced using the Illumina Hiseq 1500 sequencer platform (Annoroad, China). The filtered reads were assembled into large contigs using IDBA-ud-1.0.9 (Peng et al., 2012) and CLC Bio Genomics Workbench v10.1 (QIAGEN, Denmark). The complete genome of each phage was completed and manually inspected. ORFs encoded by the complete genome sequences were predicted by GeneMark.hmm^1^ (Besemer et al., 2001; Besemer and Borodovsky, 2005). The ORFs were annotated using the BLASTP algorithm with the non-redundant (nr) protein database of the National Center for Biotechnology Information (NCBI)^2^. tRNAs were detected using ARAGORN and tRNAscan-SE^3^ (Rombouts et al., 2016). The putative promoter sequences were predicted using the promoters online analysis tool Softberry^4^ (Bardina et al., 2016; Rombouts et al., 2016). The phage genome maps were drawn using Genome Diagram (Pritchard et al., 2006).

### Phylogenetic Analysis of the Phages

The large subunit terminase sequences of the *A. salmonicida* phages [5 phages isolated in this study and 17 phages analyzed by Vincent et al. (2017)], and other homologous sequences obtained from the GenBank database were used to construct the phylogenetic tree. Phylogenetic analysis of the large subunit terminases was carried out using MEGA 5.02^5^ (Tamura et al., 2011) with the neighbor-joining method and 1,000 bootstrap replications (Som and Fuellen, 2009). Multiple sequence alignments of the phage genomes were carried out using EasyFig^6^ (Kim et al., 2012a).

### Adsorption Curve

Phage adsorption curves were generated based on the method reported by Mateus et al. (2014), with some modifications. Briefly, phage suspensions (10 μl, final concentration 10^8^ PFU/ml) were added to the cultures (10 ml) of *A. salmonicida* (MF663675) cells (OD_600_ = 0.5) at a multiplicity of infection (MOI) of 0.001, and the mixtures were incubated at 30°C for 8–12 min. Next, 100 μl of the mixture was collected at specific time points: 0, 1, 2, 3, 4, 5, 10, 20, and 30 min, and diluted immediately with 900 μl of cold LB medium. After centrifugation (12,000 × *g*, 2 min), the supernatants were titrated to determine the number of un-adsorbed phages.

### One-Step Growth Curves

To determine one-step growth curves of the phages, 6 ml of *A. salmonicida* (MF663675) cells (OD_600_ = 0.4–0.5) was centrifuged at 8,000 × *g* for 5 min. The cell pellets were re-suspended in 300 μl of LB medium, then 600 μl of phage suspensions were added to yield an MOI of 0.01. After adsorption for 5 min at 30°C, the mixtures were centrifuged at 12,000 × *g* for 10 min to remove any un-adsorbed phage particles prior to re-suspending the samples in 6 ml of LB medium. The samples were incubated at 30°C, and aliquots were collected at 10 min intervals over a 90 min period (120 min for phage AS-sw). These aliquots were immediately diluted, and phage titers were determined using the double layer agar method (Pereira et al., 2011).

### Bacteriophage Killing Curves and *In Vitro* Phage Cocktail Design

Bacterial inactivation was determined individually for each phage (AS-szw, AS-yj, AS-zj, AS-sw, and AS-gz) and for various phage cocktail preparations. The optimal MOI was selected based on the killing curves of each phage at different MOIs (0.01, 0.1, 1, and 10) against *A. salmonicida* (MF663675). Each phage was used at the same concentration (10^6^ PFU/ml), and the phage cocktails consisting of two, three, four, and five phages were combined and evaluated, respectively. The killing curves of the different phage cocktails against the host *A. salmonicida* (MF663675), with the MOI of 0.01, were determined. Briefly, an aliquot of a single phage or a phage cocktail was mixed with the host *A. salmonicida* (MF663675) (10^8^ CFU/ml) at the predetermined MOI and then evaluated using a Growth Curve instrument (BioScreen C, 110001-892, Japan) to monitor the OD_600_ changes. Data were collected at 5 min intervals for 80 h at 30°C. For each assay, three control samples were included: the bacterial control (BC) was inoculated with the host bacteria only, and the phage control (PC) and the phage cocktail control (CB) were inoculated with phage(s) only. Samples were mixed with the host bacteria and incubated under the same conditions. For all phage infection assays, the initial titer of each phage was previously determined using the double agar layer method (Pereira et al., 2011). Three independent experiments were performed for each phage and cocktail.

### Stability Assays of Individual Phages and Phage Cocktails

We evaluated the stability of the phages and the phage cocktails efficiency when facing various environmental factors including pH and temperature according to the methods described by Li et al. (2016). Briefly, for temperature (4–80°C) and pH (2–12) treatments, filter-sterilized phage samples (10^8^ PFU/ml) were incubated at the indicated conditions for 45 and 120 min, respectively. Phage titers and bacterial inactivation before and after bacterial exposure to phages at the indicated conditions were determined using the double layer agar method (Pereira et al., 2011) and the killing curves, respectively.

### Statistical Analysis

Statistical analyses were performed using SPSS (SPSS 20.0 for Windows, SPSS Inc., United States). The existence of significant differences among the different conditions was assessed by Chi-square test. For each experimental set, the significance of the differences was assessed by Chi-square test. Three independent assays were carried out for each assay. A value of *p* < 0.05 was considered to be statistically significant.

### Accession Numbers

The complete genome sequences of the following phages have been submitted to GenBank: AS-szw, AS-yj, AS-zj, AS-sw, and AS-gz. Their accession numbers were MF498773, MF498774, MF448340, MF498775, and MF479730, respectively.

## Results

### Characterization of the Isolated *A. salmonicida* Phages

We isolated five bacteriophages using *A*. *salmonicida* (MF663675) as the host: AS-szw, AS-yj, AS-zj, AS-sw, and AS-gz from the water samples in the south of China. The phages produced clear and round plaques with the diameters ranging from 0.38 to 1.2 mm, and the largest clear plaque was formed by AS-zj (Supplementary Figure S1).

**FIGURE 1:**
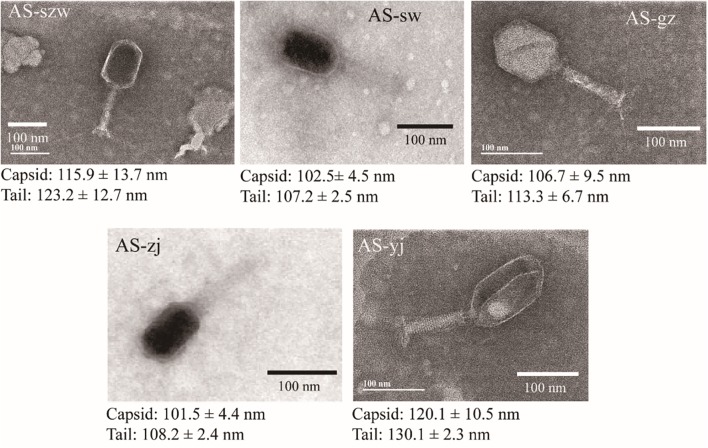
Morphologies of phages AS-szw, AS-yj, AS-zj, AS-sw, and AS-gz. The phages were stained with 2% phosphotungstic acid and visualized at 120,000× magnification with transmission electron microscopy. Scale bars represent 100 nm.

Transmission electron microscopy analysis of the phages showed that all five are morphologically similar to the phages of the *Myoviridae* family (**Figure 1**). The phages have icosahedric heads that are larger than 100 nm in diameter. AS-yj is the largest, with a 120 nm icosahedric head and a 130 nm contractile tail.

**FIGURE 2:**
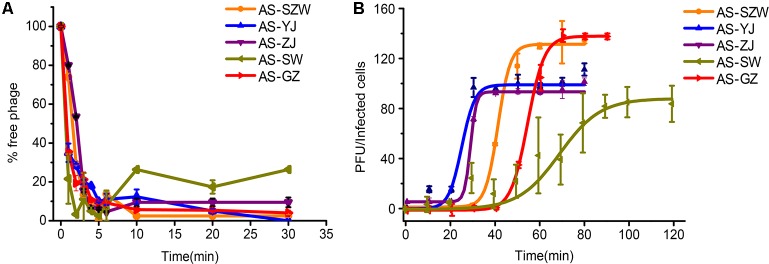
The absorption and one-step growth curves of phages AS-szw, AS-yj, AS-zj, AS-sw, and AS-gz. **(A)** The adsorption curves of the phages. The phages were grown in an exponential-phase culture of *A. salmonicida* strain (MF663675). The *y*-axis shows the percentage of free phage particles at different time points. **(B)** One-step growth curve of the phages. The phages were grown in an exponential-phase culture of *A. salmonicida* strain (MF663675). The *y*-axis shows the percentage per infected cell at different time points. Each point represents the mean value ± SD of three replicate experiments.

All the five phages showed strong adsorption to the surface of *A. salmonicida* (MF663675), and approximately 90% (2.7 × 10^4^ PFU/ml out of 3.1 × 10^4^ PFU/ml) of the phage particles adsorbed to *A. salmonicida* (MF663675) within the first 5 min (**Figure 2A**). One-step growth curves determined in LB medium at 30°C (**Figure 2B**) showed that they have different patterns of one-step curves, suggesting that they have distinct genotypes. Among the five phages, AS-yj has the shortest latency time (less than 20 min), followed by AS-zj (20 min), AS-gz (30 min), AS-szw (40 min), and AS-sw (50 min). The phage AS-szw has the highest burst size (145 PFU/host), followed by AS-gz (135 PFU/host), AS-yj (98 PFU/host), AS-sw (86 PFU/host), and AS-gz (86 PFU/host) (**Figure 2B**).

We also determined the host range of the five phages against a panel of 38 bacterial strains including 14 *Aeromonas* sp. strains (**Table 1** and Supplementary Table S1). The five phages showed the ability to lyse all the tested *A. salmonicida* strains. Additionally, phages AS-zj, AS-sw, and AS-gz have lytic capabilities against *A. hydrophila* (KF769535.1), and AS-zj has lytic activity against *A. hydrophila* (KJ806398.1). None of the phages can infect other 29 strains from the genera other than *Aeromonas*. We further typed the *A. salmonicida* strains using the RAPD and ERIC PCR primers, and the result showed different patterns of the PCR products (Supplementary Figure S2), reflecting that these *A. salmonicida* strains have variations in their genomes. Plus, we collected these bacterial strains from different geographical locations in China. All these observations suggest that these strains belong to different *A. salmonicida* subspecies. Additionally, strains YK and BG are likely psychrophilic *A. salmonicida* subsp. *salmonicida* according to the report (Long et al., 2016). Both strains were incubated at 18°C, and can grow at 4, 25, and 30°C. Meanwhile, we tested infectivity of the phages (AS-yj and AS-gz) on these two strains incubated at 18°C, and the result indicated that the phages could also form plaques on these two psychrophilic strains (Supplementary Figure S3). Our results demonstrate that the phages we isolated have high ability in infecting various *A. salmonicida* subspecies, suggesting promising in applications to treat the infections caused by *A. salmonicida* pathogens. Meanwhile, susceptibility test of *A. salmonicida* YK and BG showed that these two strains have a broad antibiotic resistant spectrum. Both can be resistant to 11 antibiotics and are only susceptible to vancomycin (YK and BG), streptomycin (YK and BG), penicillin (YK and BG), sulfamethoxazole (YK and BG), and gentamicin (YK) (Supplementary Table S2).

### Genomic Characterization and Phylogenetic Analysis of the Five Phages

**FIGURE 3:**
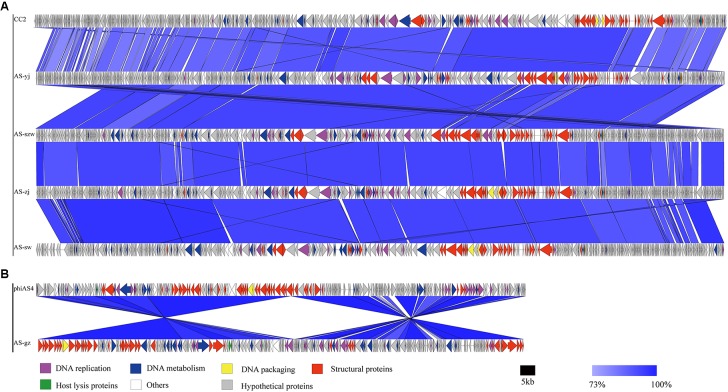
Multiple sequence alignment of phage genomes. The whole genomes of phages CC2 (GenBank accession number JX123262.1), AS-szw, AS-yj, AS-zj, AS-sw, and phiAS4 (GenBank accession number HM452125.1), and AS-gz were compared at the DNA level using Easyfig. **(A)** Genome comparison between phage AS-szw, AS-yj, AS-zj, AS-sw, and phages CC2 (GenBank accession number JX123262.1). **(B)** Genome comparison between AS-gz and phage phiAS4 (GenBank accession number HM452125.1). The blue regions indicate high sequence similarity between the genomes. The predicted function proteins are indicated by arrows with different colors.

The genomes of the five phages were sequenced and characterized (**Table 2**). Each of the five phages has linear double-stranded DNA. The GC content of the genomes ranges between 38 and 42%. The average genome size of AS-szw, AS-yj, AS-zj, and AS-sw is 230,046 (±83,217) bp and AS-gz has the smallest genome (162,475 bp). In total, 1891 ORFs were predicted, with an average of 378 ORFs per phage genome. The predicted proteins corresponding to ORFs were compared to the nr database of NCBI. In total, 84% (1488/1860) of the ORFs shared amino acid similarity (25–100%) with the sequences deposited in GenBank. The ORFs encoding putative functional proteins were assigned to the following clusters (**Figure 3**): DNA replication (e.g., helicase, DNA polymerase, ATP-dependent DNA ligase), DNA metabolism (e.g., phosphoribosyl pyrophosphate synthetase, thymidylate synthase, and ribonucleotide reductase), DNA packaging (large subunit terminase), structural proteins (e.g., tail fiber proteins, baseplate component, tail tape measure protein, and major capsid protein), host lysis (lysozyme and cell wall hydrolase protein), and others (e.g., membrane protein, inhibitor of prohead protease gp21, and inhibitor of host transcription). None of the known lysogenic-related genes was found within their genomes; therefore, we assumed all five are lytic phages. Homology-based searches using the predicted protein sequences did not identify any genes predicted to encode toxins, virulence factors, or proteins associated with antibiotic resistance, implying that these phages can be used as antibiotic agents against *A. salmonicida* infections but further investigations into the safety of the phages by fish challenges are needed before application.

**Table 2:** Phage genome characteristics.

Phage name	Genome length (bp)	GC content (%)	ORFs	Hypothetical protein	Putative protein	Bacterial promoters	tRNAs
AS-szw	229957	38.73	409	312	7	43	9
AS-yj	230183	38.80	418	313	7	46	9
AS-zj	230023	38.71	412	312	7	34	8
AS-sw	230024	38.96	414	306	5	50	9
AS-gz	162475	41.13	238	90	45	40	19

**FIGURE 4:**
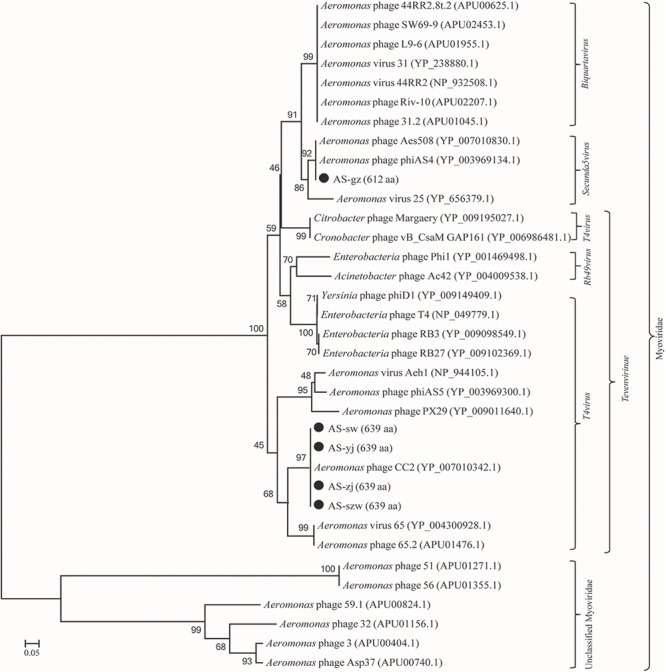
Phage phylogenetic tree based on large subunit terminases. Large subunit terminases were aligned using the Mega 5.05 program, and the phylogenetic tree was generated using the neighbor-joining method with 1,000 bootstrap replications.

Genome comparison using Easyfig suggested that the genomes of the five phages highly resemble to those of the previously characterized *Aeromonas* phages [50; 53]. As shown in **Figure 3A**, the genomes of AS-szw, AS-yj, AS-zj, and AS-sw share a high similarity with that of *Aeromonas* phage CC2 (JX123262.1). The similar virion dimensions and genome sizes between AS-gz and *Aeromonas* phage phiAS4 (HM452125.1) support their close relationship (**Figure 3B**). Phylogenetic analysis was performed based on the large subunit terminases identified in the genomes of AS-szw, AS-yj, AS-zj, AS-sw, AS-gz, as well as other reported phages (Vincent et al., 2017). The phylogenetic tree indicates that phages AS-szw, AS-yj, AS-zj, and AS-sw are closely related to the *Aeromonas* phages of the *T4virus* genus within the *Tevenvirinae* subfamily, and AS-gz clusters with another *Aeromonas* phages (Aes508, phiAS4, 25) of the *Secunda5virus* genus (**Figure 4**). They are all members of the *Myoviridae* family. AS-szw, AS-yj, AS-zj, and AS-sw are in one clade with *Aeromonas* phage CC2 (JX123262.1). CC2 was reported to efficiently infect several virulent strains of *A. hydrophila* from diseased fish (Shen et al., 2012). Phages phiAS4 (HM452125.1), Aes508 (JN377894.1), and 25 (DQ529280.1) are phages infecting strains of the species *A. salmonicida*. This finding, to some extent, explains the cross-infection of phages AS-zj, AS-sw, and AS-gz between *A. hydrophila* and *A. salmonicida* strains. Phages 59.1, 56, 32, 65.2, Asp37, 51, Riv-10, L9-6, 31.2, SW69-9, and 44RR2.8t are specific to infect psychrophilic *A. salmonicida* strains, and phage 3 as well as phages AS-yj and AS-gz are capable of infecting both mesophilic and psychrophilic *Aeromonas* strains (Vincent et al., 2017). According to their distributions on the phylogenetic tree, there are no obvious distinctions between the phages infecting psychrophilic and mesophilic strains.

### Determination of the Optimal Multiplicity of Infection (MOI)

**FIGURE 5:**
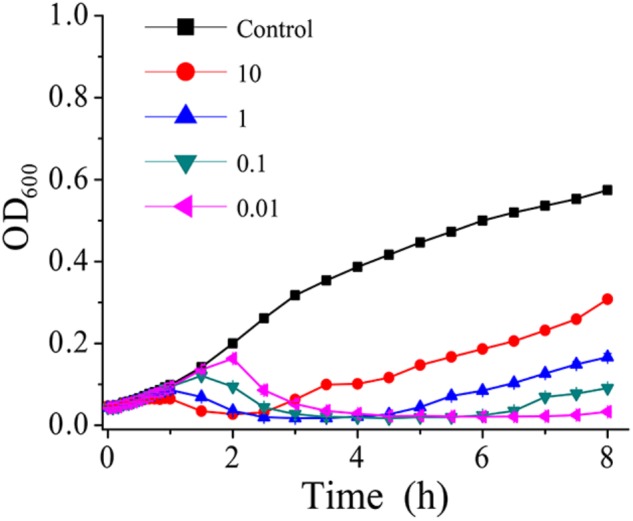
*In vitro* bacterial lytic activities of phage AS-gz at various MOIs. Killing curves of *A. salmonicida* strain (MF663675) by phage AS-gz at MOIs of 10, 1, 0.1, and 0.01 for 8 h. Each point represents the means ± SD of three replicate experiments.

Phage AS-gz was firstly selected to treat *A. salmonicida* (MF663675) using a series of MOIs (0.01, 0.1, 1, 10) (**Figure 5**) and the killing curves were determined. In **Figure 5**, the OD_600_ increased after 2.5 h at a MOI of 10, implying that heavy phage loads likely accelerated the occurrence of bacterial resistance to the phage infection in a short period of time. Notably, the killing curves indicated that AS-gz was highly effective against the host at all MOIs tested in this study. As seen from the killing curves, the efficacy of the phage load at MOIs of 0.01 was more effective than those of other MOIs against the host, which is evident by the shortened latency time and the inhibited host growth for the remaining duration of the experiment after 6 h (**Figure 5**).

### *In Vitro* Phage Cocktail Design

**FIGURE 6:**
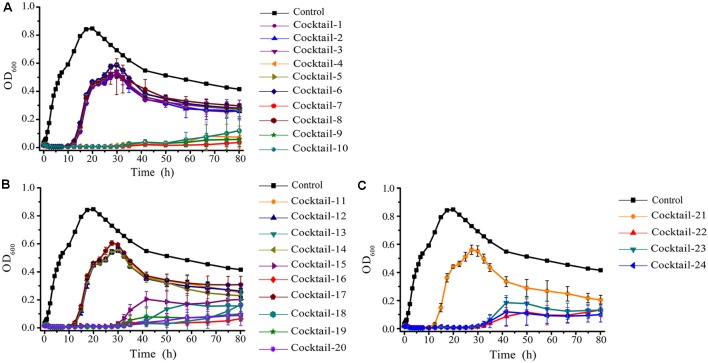
The bacterial lytic activities of the phage cocktails against *A. salmonicida* strain (MF663675). According to random permutation and combination, twenty-four cocktails were designed using phages (AS-szw, AS-yj, AS-zj, AS-sw, and AS-gz). **(A)** Cocktails with two phages; **(B)** cocktails with three phages; and **(C)** cocktails with four and five phages. Phage combinations in each cocktail were described in Supplementary Table S3. All of the phages in each cocktail were plated in Luria-Bertani agar and overlain with a liquid culture of *A. salmonicida* (MF663675). The plates were incubated at 30°C. Clear, well-defined plaques were observed and counted after 12 h.

The emergence of phage-resistant bacterial variants was observed shortly after treatment with only a single phage (**Figure 5**), and can be theoretically overcome by employing phage cocktails composed of multiple phages. Based on the characters of the isolated phages (AS-szw, AS-yj, AS-zj, AS-sw, and AS-gz), we designed a combinatorial series of phage cocktails and investigated their ability in controlling *A. salmonicida* cells at the optimal MOI of 0.01. The bacterial killing curves of the cocktails composed of AS-yj, AS-szw, AS-zj, or AS-sw appeared to have not more significant advantages in controlling bacteria growth as we can see from **Figure 6** that their latency times were 10 h and the bacterial cells grew up to 0.6 of OD_600_ within 30 h. We observed high performance of the cocktails containing the phage AS-gz in bacteria growth inactivation studies. The bacterial cell density remained less than 0.2 (OD_600_) throughout the duration of the experiment (80 h), suggesting that AS-gz likely played a dominant role in bacterial growth control when it was part of the cocktails. Particularly, slight differences of the killing curves also can be observed among the cocktails containing AS-gz. Notably, the cocktail comprised of AS-yj and AS-gz maintained its bacterial inactivation with an OD_600_ < 0.05 for 80 h (Supplementary Figure S4). In addition, the cocktail was also tested against the panel of 38 bacterial strains, and the result showed that the host range of the cocktail was a superposition between AS-yj and AS-gz (**Table 1** and Supplementary Table S1). These results demonstrated that the cocktail composed of As-yj and AS-gz is significantly promising to be used in controlling *A. salmonicida* infections.

### Stability of the Phages

**FIGURE 7:**
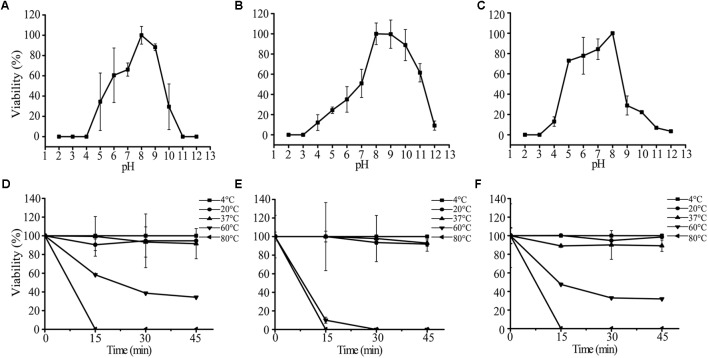
Stability analysis of AS-yj, AS-gz, and the cocktail of AS-yj and AS-gz. **(A–C)** Influences of pH after incubation for 120 min in LB broth at 30°C. **(D–F)** Influences of temperature on the phages AS-yj and AS-gz, and the cocktail of AS-yj and AS-gz at 4, 20, 37, 60, and 80°C, respectively. Samples were collected at 15 min intervals for 45 min at each temperature. The data were the phage titers and determined using the double layer agar method. Each point represents the mean value ± SD of three replicated experiments.

We tested the impacts of pH and temperature on the infectivity of AS-gz, AS-yj, and the cocktail composed of AS-gz and AS-yj. Phages AS-yj, AS-gz, and the cocktail were stable over a wide pH range (**Figure 7**). Plaque counting showed that AS-yj and AS-gz maintained their lytic capacity at pH 5–10 (**Figure 7A**) and 4–11 (**Figure 7B**), respectively. Interestingly, the cocktail displayed relatively greater stability in lytic capacity with its infectivity over 80% at pH 5–8 (**Figure 7C**) than either of the individual phages (Supplementary Figure S5). The activities of the phages and the cocktail were not influenced by temperatures between 4 and 37°C (**Figures 7D–F**). It was observed that the high temperature treatment inactivated 66% (resulting in 6.57 × 10^7^ PFU reduction) and 100% (10^8^ PFU reduction) of AS-yj at 60 and 80°C, respectively (**Figure 7D**). Phage AS-gz appeared to be more sensitive to high temperature, with approximately 90% (9.1 × 10^7^ PFU reduction) decrease in titer observed after incubation for 15 min at 60°C, and it was completely inactivated at 80°C for 15 min (**Figure 7E**). Although it seems that the cocktail, with a combination of AS-yj and AS-gz, has not more significant advantages than the individual phages as shown in **Figure 7F**, which was drawn according to phage particle counting, evaluation according to bacterial inactivation determined by the killing curves still revealed that the cocktail (AS-yj and AS-gz) displayed a stronger lytic ability than individual phages with OD_600_ less than 0.2 (Supplementary Figure S6). Taken together, these results suggest that there may be some synergies between these two phages in infecting the host.

## Discussion

With the emergence of multidrug-resistant bacterial pathogens in both clinical and aquaculture settings, a growing number of studies implied that phages are a potential and promising alternative solution in controlling multidrug-resistant pathogens (Kim et al., 2012b; Chan et al., 2013; Jaiswal et al., 2013; Mateus et al., 2014; Mapes et al., 2016). In the present study, we isolated five phages from the water samples collected from different locations in Guangdong province, China. Further, we characterized them by determining their adsorption abilities, one-step curves, morphological features, and genomic sequences. A panel of 38 various bacterial strains isolated from different geographic locations with great distances were used to determine the host range of AS-szw, AS-yj, AS-zj, AS-sw, and AS-gz, and the result showed that they are able to efficiently infect a range of *A*. *salmonicida* strains which were confirmed belonging to different subspecies of *A. salmonicida* by RAPD-PCR and ERIC-PCR analyses. Thus, the phages we isolated in this study are promising antimicrobial agents that can be used in treating *A. salmonicida* infections in aquaculture.

It is of interest to observe that phages AS-yj and AS-gz were capable of infecting strains BG and YK at 30 (**Table 1**) and 18°C (Supplementary Figure S3) as well, although BG and YK are likely psychrophilic *A. salmonicida* subsp. *salmonicida* strains (Long et al., 2016). The phages we isolated from the samples of mesophilic environments showed infectivity on strains BG and YK at 30°C. It has been known that incubating *A. salmonicida* ssp. *salmonicida* at temperatures above 25°C may cause many disturbances in the genome (Chu et al., 1995; Stuber et al., 2003). The disturbances could affect the *vapA* gene coding for the A-layer, which is a potential receptor for phage infection (Long et al., 2016; Vincent et al., 2017). Interestingly, our results indicated that the phages can infect these two strains at both 18 and 30°C, reflecting the infection mechanisms employed by phages are diverse in nature, and there likely exist some other potential receptors for the phages targeting *A. salmonicida* ssp. *salmonicida* in this study (Pavan et al., 2000; Vincent et al., 2017), which deserves further investigations. Additionally, antibiotic susceptibility test of strains YK and BG were carried out on 24 antibiotics at 18°C, and the results showed that these two strains have a broad antibiotic resistant spectrum, verifying that phage infection on their host strains is irrespective of their host resistance to antibiotic (Pirnay et al., 2011; Supplementary Figure S3).

Multiplicity of infection is an important parameter when phages are applied to challenge bacteria. In this study, it is of interest to observe that phage-resistant bacterial variants may be induced by heavy phage load more rapidly than those treated with low load. As shown in **Figure 5**, the bacterial density (OD_600_) rapidly increased when infected at higher MOIs (10 and 1), although the bacteria cells were inactivated earlier than at lower MOIs (0.1 and 0.01). Comparing of the killing curves of the phages against the host at MOIs of 0.1 and 0.01 resulted in the optimal MOI as 0.01 that was used in next step experiments. This observation also suggests that heavy phage loads likely result in the emergence of bacterial resistance to the phage infection. As reported by Castillo et al. (2015), bacterial population can evolve rapidly from the dominance of phage-sensitive clones to phage-resistant clones when exposed to the selection pressure of lytic phages (Middelboe, 2000; Middelboe et al., 2001; Hyman and Abedon, 2010; Castillo et al., 2015). This information indicates that heavy phage loads perhaps is a selection pressure and should be taken into consideration in phage therapy.

In practice, it was inevitable to observe that phage-resistant variants rapidly emerge when a single phage type was used to control specific pathogens (Chan et al., 2013; Barbu et al., 2016). In this study, we also observed that the infection with phage AS-gz resulted in the rapid increases of OD_600_ at an early time points (**Figure 5**). Aiming to broaden host-range, Mapes et al. (2016) developed a procedure for cocktail formulation involving multiple passages on previous phage-resistant strains. Similarly, Gu et al. (2012) developed an efficient phage cocktail using a step-by-step method. Kelly et al. (2011) reported to directly use phages isolated from environment, according to their host spectrum, to develop a broad-host-range phage cocktail. Although many studies reported on successfully using phage cocktails as antimicrobial agents against bacterial pathogens, those cocktails were combined with phages randomly. In this study, we observed that the efficacies of the cocktails combining distinct phages varied. Bacterial inactivity tests showed that the phage cocktail comprised of AS-gz and AS-yj has the highest efficacy against the host. Conventional phage cocktails were usually designed based on the assumption that the more specific phages we include in a cocktail, the higher the efficacy of the phage cocktail against the target bacterial host is (Labrie et al., 2010; Pirnay et al., 2011; Chan et al., 2013). The result of our study demonstrated that a phage cocktail containing only two phages, AS-gz and AS-yj, had the ability to control the host growth for a longer time than other formulated cocktails (**Figure 6**). These data imply too many types of phages present in a given cocktail will cause competitive inhibition of phage infection against the host. Also, the cocktail composed of AS-gz and AS-yj has several advantages, such as higher efficacy in inactivating their hosts, higher stability under different temperature, and pH than any of the individual phages. In particular, we noted that phage AS-gz has a genome sequence that is greatly different from those of the other four *A. salmonicida* phages, suggesting that AS-gz likely uses a different infection mechanism against the host from the others, highlighting the importance of the knowledge obtained from genome sequencing prior to phage cocktail preparation.

In our study, we observed that phage-resistant variants rapidly emerged (**Figure 6**). The rapid emergence of phage-resistant bacteria is the main concern for phage therapy. It has been known that bacteria have many mechanisms to evade phage infection (Labrie et al., 2010). CRISPR system is one of them, but only can be found in 40% bacteria genomes (Labrie et al., 2010). We investigated all (in total 42) sequenced *A. salmonicida* genomes [including five strains (YK, BG, S121, S44, and S68) we used in this study] using the software CRISPRfinder online^7^ (data not shown). We did not find any CRISPR or CRISPR-like system in the genomes. Thus, we speculate here that other mechanisms that likely helped the *A. salmonicida* strains evade the phage infection, such as: (1) preventing phage adsorption, which includes blocking of phage receptors (*Staphylococcus aureus* et al.), the production of extracellular matrix (*Pseudomonas* spp. et al.), and the production of competitive inhibitors (*Escherichia coil*); (2) preventing phage DNA entry, which depends on different superinfection exclusion (Sie) systems (*Lactococcus lactis* et al.); (3) cutting phage nucleic acids, including restriction–modification (R–M) systems (*Streptococcus pneumoniae*) and the CRISPR-Cas system (*P. aeruginosa*); and (4) abortive infection (Abi) systems, which target the crucial step of phage multiplication including replication, transcription, or translation (Labrie et al., 2010). Due to the diverse and complicated mechanisms that bacteria have against phage infection, it is unlikely to determine the exact mechanism in this study. Based on our observation that the tested strains evolved with resistance against phage infection rapidly, it is likely that *A. salmonicida* strains changed the structure of their cell surface receptors or their three-dimensional conformation to get resistance to phage infection. Like *S. aureus* (Nordström and Forsgren, 1974; Foster, 2005), *A. salmonicida* cells might produce cell-wall-anchored virulence factors to mask the receptors. In addition, the production of structured extracellular polymers by *A. salmonicida* can provide a physical barrier, which prevents phages from binding to *A. salmonicida* receptors (Sutherland, 1999; Stuber et al., 2003; Stummeyer et al., 2006).

## Conclusion

We aimed to develop an alternative antimicrobial agent against multidrug-resistant *A. salmonicida*. Five *A. salmonicida* phages were isolated and characterized. Accordingly, phage cocktails were designed *in vitro* and investigated on the hosts. Our data manual confirmed that the cocktail comprised of phages AS-gz and AS-yj showed promising and potential to be an alternative agent to fight the multidrug-resistant pathogen *A. salmonicida*. Further studies regarding the *in vivo* assays will be carried out to determine the efficacy of the phage cocktail (AS-yj and AS-gz) for bio-control of *A. salmonicida* infections in aquaculture environments.

## Author Contributions

LC and YM conceived and designed the experiments. LC and SY performed the experiments. LC, QL, and GM analyzed the data. JY and DD contributed sample materials. SY and YM wrote the paper. YM, CL, BZ, and CL managed the project.

## Conflict of Interest Statement

The authors declare that the research was conducted in the absence of any commercial or financial relationships that could be construed as a potential conflict of interest.

## Footnotes

1 http://exon.gatech.edu/GeneMark/index.html

2 http://www.ncbi.nlm.nih.gov

3 http://lowelab.ucsc.edu/tRNAscan-SE/

4 http://www.softberry.com/berry.phtml

5 http://www.megasoftware.net

6 https://mjsull.github.io/Easyfig/

7 http://crispr.i2bc.paris-saclay.fr/Server/
