# Supplementary material for: In Vitro Design and Evaluation of Phage Cocktails Against Aeromonas salmonicida
Source: Front Microbiol. 2018 Jul 6;9:1476. doi: 10.3389/fmicb.2018.01476 (PMC6043867; doi:10.3389/fmicb.2018.01476)
Supplement: Supplementary file 1 [file Presentation_1.PDF]

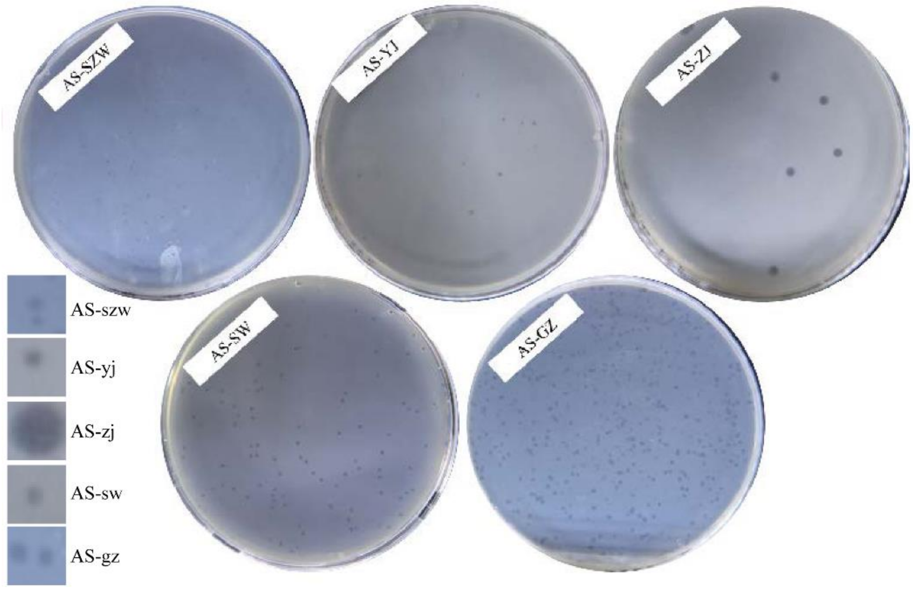


Figure S1. Morphology of plaques of AS-szw, AS-yj, AS-zj, AS-sw, and AS-gz, respectively.

Phages were plated in Luria-Bertani agar and overlain with a liquid culture of *Aeronomas salmonicida* (MF663675.1). The plates were incubated at 30°C. Clear, well-defined plaques were observed and photographed after 12 h. The magnified plaques of each phage were on the left down and on the same magnification.

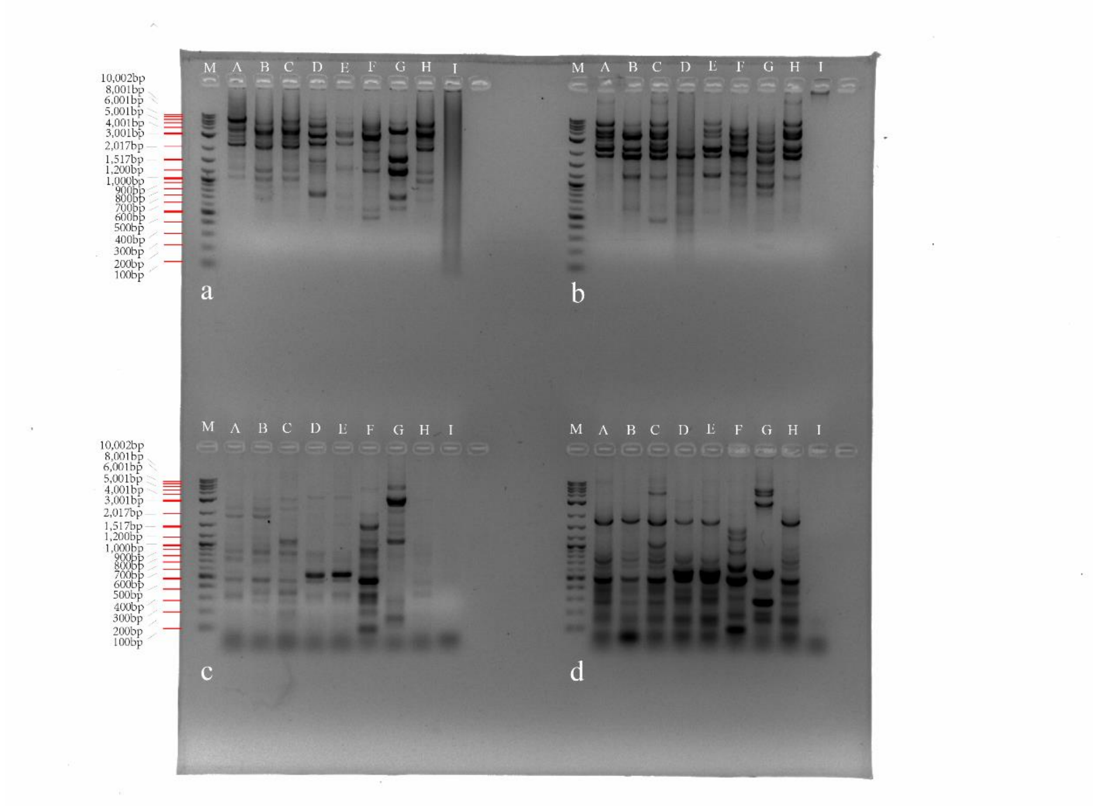


**Figure S2**. Gel electrophoresis profiles of the products obtained from RAPD-PCR and ERIC-PCR of *Aeromonas salmonicida* strains of as list in Table 1.

In each lane illustrates the DNA marker (100 bp-10,000bp), *A. salmonicida* S44 (CP022181.1), *A. salmonicida* S68 (CP022186.1), *A. salmonicida* S121 (CP022175.1), *A. salmonicida* YK (KC254649.1), *A. salmonicida* BG (KC254648.1), *A. salmonicida* (MF632072), *A. hydrophila* (MF663676), *A. salmonicida* (MF663675), control. a: primers AP5, 5'-TCACGCTGCG-3'; b: primers OPB-7, 5'-GGTGACGCAG-3'; c: AP3- 5'-TCACGATGCA-3'; d: ERIC-1R, 5'-ATGTAAGCTCCTGGGGATTCAC-3', ERIC 2, 5'-AAGTAAGTGACTGGGGTGAGCG-3'

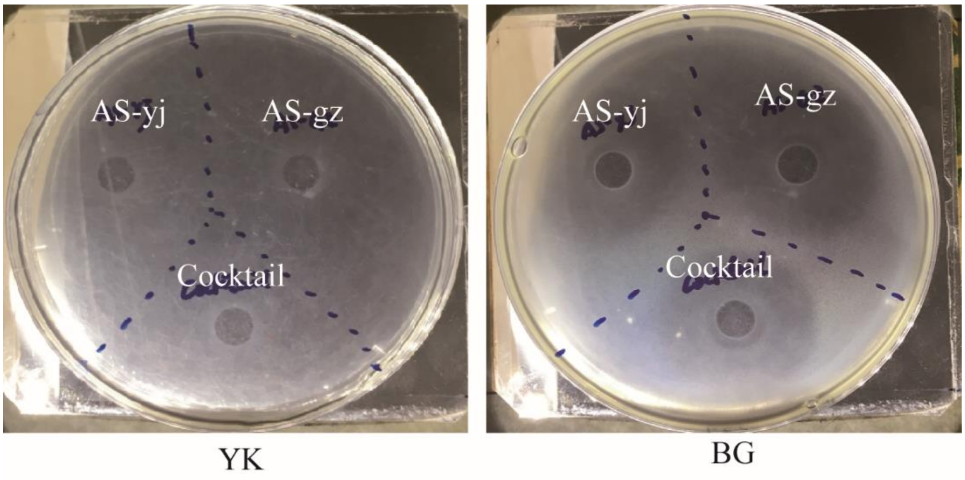


**Figure S3**. Morphology of lytic cycles of phages AS-yj, AS-gz, and cocktail (AS-yj and AS-gz) on *Aeronomas salmonicida* YK and BG.

Phages were plated in Luria-Bertani agar and overlain with a liquid culture of *Aeronomas salmonicida* YK and BG. The plates were incubated at 18°C. Clear, well-defined lytic plaques were observed and photographed after 12 h.

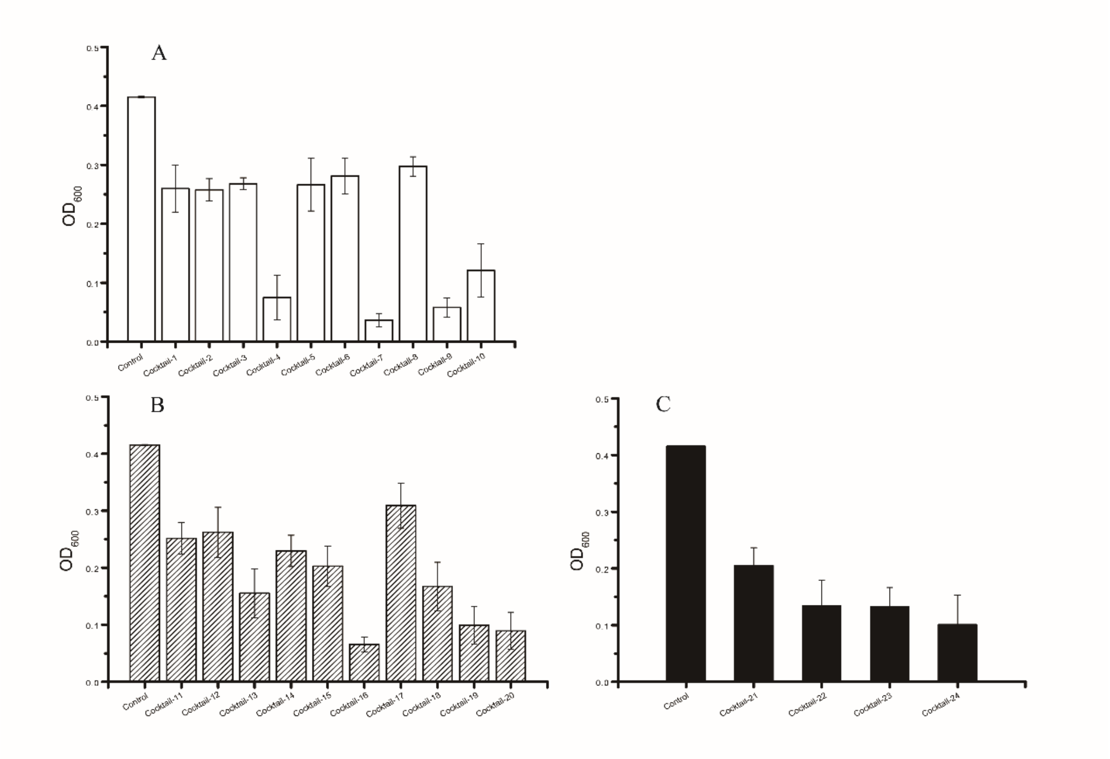


**Figure S4**. The final $OD_{600}$ of the host bacteria after infected by each cocktail for 80 h, the legend refered to Fig. 6.

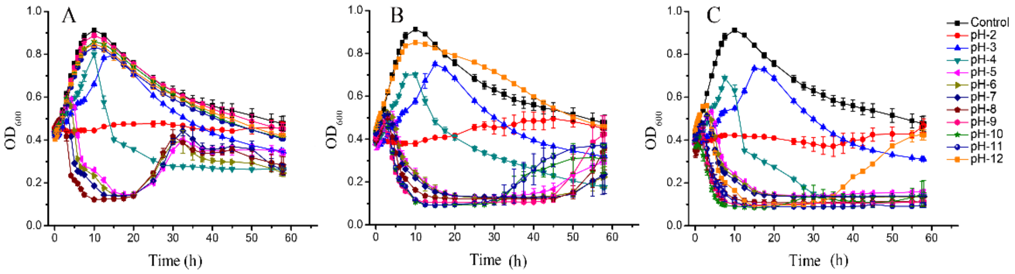


Figure S5. Killing curves of AS-yj, AS-gz and cocktail (AS-yj and AS-gz).
A, B, ans C: influence of pH after incubation for 120 min in LB broth at 30°C on the bacterial inactivation of the phages AS-yj, AS-gz, and cocktail (AS-yj and AS-gz), respectively. The range of pH was set from 2 to 12. Each point represents the mean value ± S.D. of three replicated experiments.

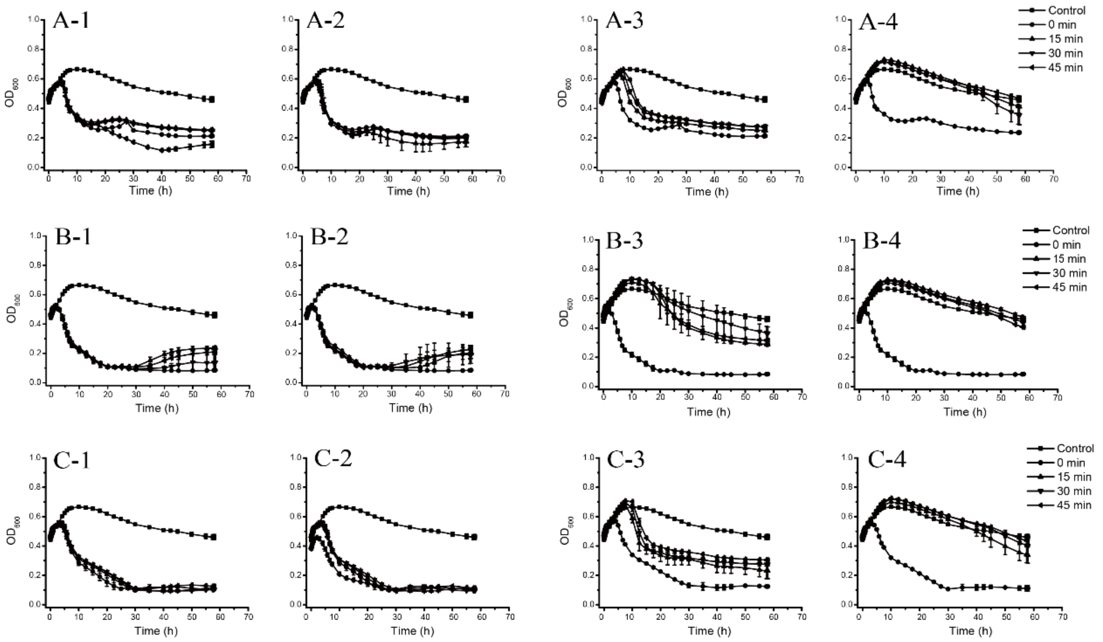


Figure S6. Effects of temperature on the bacterial inactivation of AS-yj, AS-gz and cocktail (AS-yj and AS-gz).

A (A-1, A-2, A-3, and A-4), B (B-1, B-2, B-3, and B-4), C (C-1, C-2, C-3, and C-4): influence of temperature on the phages AS-yj, AS-gz and cocktail (AS-yj and AS-gz) at 20°C, 37°C, 60°C, and 80°C, respectively. Samples were collected at 15 min intervals for 45 min at each temperature, and combined with the exponential stage host culture respectively. Each point represents the mean value ± S.D. of three replicated experiments.

Table S1. Phage lytic spectra on the bacterial strains used in this study

| Species | ID | Infectivity | | | | | |
|---|---|---|---|---|---|---|---|
| | Accession | AS-szw | AS-yj | AS-zj | AS-sw | AS-gz | Cocktail7 |
| *Aeromonas salmonicida* strain* | MF663675.1[e] | + | + | + | + | + | + |
| *A. salmonicida BG* strain[a] | KC254648.1[f] | + | + | + | + | + | + |
| *A. salmonicida YK* strain[a] | KC254649.1 [f] | + | + | + | + | + | + |
| *A. salmonicida S121* strain[b] | CP022175.1[f] | + | + | + | + | + | + |
| *A. salmonicida S68* strain[b] | CP022186.1[f] | + | + | + | + | + | + |
| *A. salmonicida S44* strain[b] | CP022181.1[f] | + | + | + | + | + | + |
| *A. salmonicida* strain[c] | MF632072[e] | + | + | + | + | + | + |
| *A. hydrophila* strain[d] | MF663676[e] | - | - | + | - | - | - |
| *A. hydrophila* strain[d] | MF663672[e] | - | - | + | + | + | + |
| *A hydrophila* strain | AY987739.1 | - | - | - | - | - | - |
| *A hydrophila* strain | KJ806398.1 | - | - | - | - | - | - |
| *A hydrophila* strain | KT363959.1 | - | - | - | - | - | - |
| *A. caviae* strain | NZ_LAFH0100002 | - | - | - | - | - | - |
| *A. veronii* strain | NZ_MRZQ01000043 | - | - | - | - | - | - |
| *Bacillus cereus* strain | KX783593.1 | - | - | - | - | - | - |
| *B subtilis* strain | KX783604.1 | - | - | - | - | - | - |
| *Clostridium beijerinckii* strain | KJ957170.1 | - | - | - | - | - | |
| *Enterobacter faecalis* strain | HQ802157.1 | - | - | - | - | - | - |
| *E faecium* strain | KP137385.1 | - | - | - | - | - | - |
| *E.coli* strain | KP789331.1 | - | - | - | - | - | - |
| *Klebsiella pneumoniae* strain | CP019219.1 | - | - | - | - | - | - |
| *K pneumoniae* strain | KJ803925.1 | - | - | - | - | - | - |
| *K variicola At-22* strain | CP010523.2 | - | - | - | - | - | - |
| *Lactobacillus fermentum* strain | KU213665.1 | - | - | - | - | - | - |
| *L plantarum* strain | CP017406.1 | - | - | - | - | - | - |
| *L plantarum* strain | KM497500.1 | - | - | - | - | - | - |
| *Pediococcus acidilactici* strain | KU504251.1 | - | - | - | - | - | - |
| *P lolii* strain | KT315923.1 | - | - | - | - | - | - |
| *Pseudomonas aeruqinosa* strain | KM491554.1 | - | - | - | - | - | - |
| *Salmonella enterica-M* strain | CP019410.1 | - | - | - | - | - | - |
| *S enterica-SL7207* strain | NZ_MPJVO1000028.1 | - | - | - | - | - | - |
| *S thermophilus* strain | CP012588.1 | - | - | - | - | - | - |
| *S thermophilus* strain | CP016394.1 | - | - | - | - | - | - |
| *Vibrio alginolyticus* strain | KT986134.1 | - | - | - | - | - | - |
| *Vibrio anguillarum* strain | FJ378038.1 | - | - | - | - | - | - |
| *Vibrio azureus* strain | JN603238.1 | - | - | - | - | - | - |
| *Vibrio mimicus* strain | CP016383.1 | - | - | - | - | - | - |
| *Vibrio natriegens* strain | EF187013.1 | - | - | - | - | - | - |

[*] strain isolated in aquaculture in South of China and used on the enrichment procedure for phage isolation;

[a] strains purchased from State Key Laboratory of Freshwater Ecology and Biotechnology, Institute

of Hydrobiology, Chinese Academy of Sciences, Wuhan;

[b] strains gained from State Key Laboratory of Microbiology Resources, Institute of Microbiology, Chinese Academy of Sciences, Beijing;

[c] strain provided by Sichuan Agricultural University (Chengdu City, Sichuan Province);

[d] strains isolated from wastewater of an aquaculture farm in Zhanjiang City, Guangdong Province

[e] 16S rRNA sequences number of the relative strains waiting to be released;

[f] Genome sequences number of the relative strains

The rest strains used for host spectrum test and marked with blue color were isolated from environment samples.

Cocktail7 is formed with phages AS-yj and AS-gz.

Table S2 Susceptibility test results to 24 antibiotics of Aeromonas salmonicida YK and BG

| Antibiotics | Diameter of antibacterial circle | |
|---|---|---|
| | YK (mm) | BG (mm) |
| chloromycetin | R | R |
| florfenicol | R | R |
| enrofloxacin | R | R |
| tetracycline | R | R |
| cefotaxime | R | R |
| pipemidic acid | R | R |
| norfloxacin | R | R |
| ofloxacin | R | R |
| ceftizoxime | R | R |
| cefazolin | R | R |
| doxycycline | R | R |
| furazolidone | S | R |
| macrodantin | R | R |
| gentamicin | I3 | I |
| rifampicin | R | R |
| erythrocin | R | R |
| neomycin | I | I |
| tobramycin | I | I |
| Kanamycin | R | I |
| Polymyxin B | R | I |
| vancomycin | S | S |
| streptomycin | S | S |
| penicillin | S | S |
| sulfamethoxazole | S | S |

R: resistant; I:intermediate; S: susceptible

Table S3 Refer to the legend of Fig. 6

| Phages formed in cocktails | | | |
|---|---|---|---|
| cocktails with two phages | | | |
| cocktail-1 | AS-szw, AS-yj | cocktail-6 | AS-yj, AS-sw |
| cocktail-2 | AS-szw, AS-zj | cocktail-7 | AS-yj, AS-gz |
| cocktail-3 | AS-szw, AS-sw | cocktail-8 | AS-zj, AS-sw |
| cocktail-4 | AS-szw, AS-gz | cocktail-9 | AS-zj, AS-gz |
| cocktail-5 | AS-yj, AS-zj | cocktail-10 | AS-sw, AS-gz |
| cocktail with three phages | | | |
| cocktail-11 | AS-szw, AS-yj, and AS-zj | cocktail-16 | AS-yj, AS-zj, and AS-sw |
| cocktail-12 | AS-szw, AS-yj, and AS-sw | cocktail-17 | AS-yj, AS-zj and AS-sw |
| cocktail-13 | AS-szw, AS-yj, and, AS-gz | cocktail-18 | AS-yj, AS-zj, and AS-gz |
| cocktail-14 | AS-szw, AS-zj, and AS-sw | cocktail-19 | AS-yj, AS-sw, and AS-gz |
| cocktail-15 | AS-szw, AS-zj, and AS-gz | cocktail-20 | AS-zj, AS-sw, and AS-gz |
| cocktail-16 | AS-szw, AS-sw, and AS-gz | | |
| cocktail with four and five phages | | | |
| cocktail-21 | AS-szw, AS-yj, AS-zj and AS-sw | cocktail-23 | AS-yj, AS-zj, AS-sw and AS-gz |
| cocktail-22 | AS-szw, AS-yj, AS-zj and AS-gz | cocktail-24 | AS-szw, AS-yj, AS-zj, AS-sw, and AS-gz |
